# Early-onset intrahepatic cholestasis of pregnancy increased the incidence of gestational diabetes mellitus: a retrospective cohort study

**DOI:** 10.3389/fmed.2024.1441085

**Published:** 2024-08-22

**Authors:** Yaodan Liu, Min Liu, Jinghua Liu, Minmin Sheng, Zhenxia Hu, Xiaohong Zhang

**Affiliations:** ^1^Department of Gynecology and Obstetrics, Shanghai Public Health Clinical Center, Fudan University, Shanghai, China; ^2^Department of Pharmacy, Shanghai Public Health Clinical Center, Fudan University, Shanghai, China

**Keywords:** intrahepatic cholestasis of pregnancy, gestational diabetes mellitus, early-onset, risk factors, cohort study

## Abstract

**Background:**

Intrahepatic cholestasis of pregnancy (ICP) and gestational diabetes mellitus (GDM) are two common pregnancy complications that pose considerable health challenges. The interplay between these conditions is believed to significantly influence pregnancy outcomes, yet the nature of this relationship remains elusive. This study was designed to elucidate the connection between ICP and GDM.

**Methods:**

This retrospective cohort study included 742 singleton pregnancies delivered at the Shanghai Public Health Clinical Center from January 2015 to December 2023. We compared the incidence of GDM and pregnancy outcomes between multiple ICP subgroups and a control group of healthy pregnancies. A multivariate regression model was used to measure the independent association between ICP and propensity for GDM development, as well as to assess the impact of potential bidirectional effects between ICP and GDM.

**Results:**

The results indicate that the incidence of GDM is highest in the early-onset ICP (diagnosed before the 24th week of gestation) group compared to the control group and other ICP subgroups. Early-onset ICP is an independent risk factor for the development of GDM, with other risk factors including age, history of abortion, family history of diabetes, and elevated ALT levels. Subgroup interaction analysis did not reveal heterogeneity in the influence of early-onset ICP on the development of GDM across different subgroups. Further analysis showed that GDM itself does not increase the risk of late-onset ICP. Additionally, when comparing pregnancy outcomes between GDM patients with or without ICP, those with both GDM and ICP had significantly higher rates of preterm birth, cesarean section, and small for gestational age (SGA) compared to patients with GDM alone. Furthermore, elevated TBA levels (first diagnosed) of early-onset ICP patients were associated with an increased risk of GDM in a nonlinear fashion.

**Conclusion:**

Our study indicated that early-onset ICP is significantly linked to an increased risk of GDM. Further research is warranted to explore the mechanisms behind this association and to develop strategies for early identification and intervention to mitigate GDM risk.

## Introduction

Intrahepatic cholestasis of pregnancy (ICP) is a serious complication that occurs in 1–27.6% of pregnancies, with a reported prevalence of 6.06% in China (1). It is characterized by pruritus, elevated serum transaminases, and increased levels of total bile acids (TBA), which poses serious risks to both maternal and fetal health. Adverse outcomes commonly associated with ICP include premature birth, respiratory distress, asphyxia, and preeclampsia (2–4).

Gestational diabetes mellitus (GDM) is another critical pregnancy complication, defined as hyperglycemia with first onset or detection during pregnancy. In China, the prevalence of GDM ranges from 12.8 to 16.7%, elderly, obese, or women with a family history of diabetes have a higher incidence rate of GDM (5). GDM can trigger a range of neonatal complications, including macrosomia, hypoglycemia, and respiratory distress. Moreover, it elevates the risk of developing type 2 diabetes in mothers postpartum (6).

Understanding the interplay between ICP and GDM is crucial, given the potential compounded risks both conditions pose to pregnancy outcomes. Despite existing research exploring the relationship between ICP and GDM, the nature of this connection remains unclear and is a subject of debate in the academic community. While several studies suggest an increased risk of GDM in patients with ICP (7, 8)—especially among those with severe cases (9, 10) or twins (11)—findings are inconsistent, a Danish cohort study found no significant association between the two (12). Additionally, since the diagnostic criteria for GDM were revised in 2010 (13), assessing the interrelationship between ICP and GDM in light of these changes is particularly relevant.

The contradictory findings in studies exploring the relationship between ICP and GDM may be attributed to several factors. First, differences in study design and methodology, such as sample selection, diagnostic criteria, and the type of study, may lead to inconsistent results. Second, variations in race and genetic background could affect the incidence of ICP and GDM and their interrelationship. Furthermore, lifestyle and environmental factors, including dietary habits, physical activity levels, and environmental exposures, may play a key role in the development of ICP and GDM, adding complexity to the research outcomes. The timing of ICP onset, whether early or late in pregnancy, and its impact on GDM development is not well understood, which further complicates the interpretation of study results.

Given the current gaps in understanding the relationship between ICP and GDM, and the inconsistencies in existing research findings, our study is designed to fill these knowledge gaps. We aim to deepen the understanding of the link between these two conditions by confirming the correlation between ICP and GDM, and by examining whether factors such as the timing of ICP onset, associated liver dysfunction, and total bile acid (TBA) levels influence the risk of GDM. Establishing a clear connection between these conditions will aid in the development of more effective management strategies for pregnant women who suffer from these complications.

## Materials and methods

**Figure 1:**
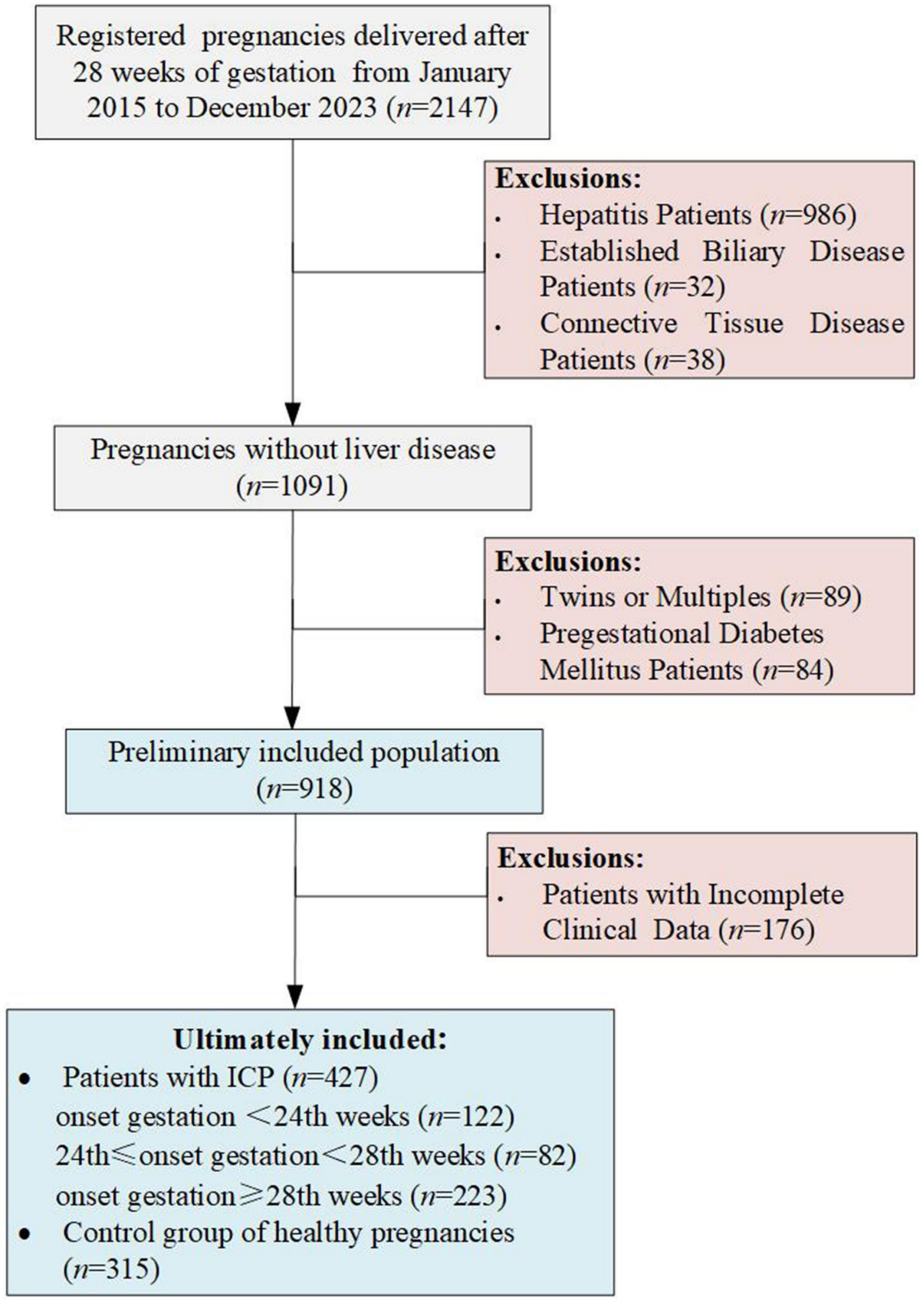
Flow chart of pregnancies selection.

This retrospective study encompassed singleton pregnancies delivered at the Shanghai Public Health Clinical Center from January 2015 to December 2023, focusing exclusively on women who reached at least 28 weeks of gestation, miscarriages prior to 28 weeks were excluded. Initially, the study considered 2047 pregnant women. Exclusion criteria were twin or multiple pregnancies, patients with comorbid hepatitis viruses including hepatitis B, C, and E, confirmed bile diseases (such as cholecystitis or gallstones), autoimmune diseases (like connective tissue diseases or systemic lupus erythematosus), pre-existing diabetes, and those with incomplete clinical or laboratory data. Ultimately, 742 pregnancies delivered after 28 weeks of gestation remained eligible for analysis (Figure 1). All cases of intrahepatic cholestasis of pregnancy (ICP) were diagnosed based on fasting serum total bile acid levels ≥10 μmol/L or postprandial serum levels ≥20 μmol/L, excluding women who exhibited itching without elevated bile acids. The diagnosis of ICP is inclusive of cases where elevated serum total bile acid levels are detected at any gestational age. In light of the diagnosis of gestational diabetes occurring after the 24th week of pregnancy, for this study, we have defined patients diagnosed with ICP prior to 24th weeks as early-onset ICP. Demographic information such as age, height, pre-pregnancy BMI, parity, history of abortion, family history of diabetes, and results of the Oral Glucose Tolerance Test (OGTT) were collected for data extraction. Gestational diabetes mellitus (GDM) was diagnosed according to the criteria set by the International Association of the Diabetes and Pregnancy Study Groups (IADPSG), identifying GDM between the 24th and 28th weeks of gestation if OGTT results exceeded fasting plasma glucose levels greater than 5.1 mmol/L, 1-h plasma glucose levels greater than 10 mmol/L, or 2-h plasma glucose levels greater than 8.5 mmol/L.

The study was approved by the ethics committee of the Shanghai Public Health Clinical Center (2022-Y061-01).

### Statistical analysis

All analyses were conducted using STATA/BE version 18.0. Descriptive statistics, including proportions, means, medians, and ranges, were computed to provide an overview of the data. Categorical variables were analyzed using the Chi-square or Fisher’s exact test, while continuous variables were compared using the T-test, to discern differences in baseline characteristics between all ICP groups and the non-ICP group, or to evaluate differences in pregnancy outcomes between all ICP groups and non-ICP groups within GDM patients. Stratified analyses were employed to identify potential confounders and effect modifiers. The Breslow-Day test was utilized to examine the homogeneity of the odds ratios (ORs) for early-onset ICP across different risk factor strata. The association between early-onset ICP and GDM was evaluated using multivariable logistic regression, with odds ratios and 95% confidence intervals (CIs) estimated accordingly. We also employed multifactorial logistic regression analysis to delve into the relationship between GDM patients and late-onset ICP. Utilizing this analytical approach, we were able to adjust for a range of potential confounding factors, thereby providing a more precise assessment of the impact of potential bidirectional effects between ICP and GDM. Variable selection for the regression models was informed by statistical and clinical significance. The relationship between TBA levels and GDM risk in early-onset ICP patients was further explored with restricted cubic spline (RCS) analysis, performed using R.4.2.3, along with Zstats v0.91.^1^ Results were reported with two-tailed *p*-values, considering values less than 0.05 as statistically significant.

## Results

Between January 2015 and December 2023, a total of 427 patients with intrahepatic cholestasis of pregnancy (ICP) and 315 control pregnancies were recruited according to the inclusion and exclusion criteria. In the cohort of patients diagnosed with ICP, 122 were diagnosed prior to the 24th week of gestation, 82 were diagnosed between the 24th and 28th weeks of gestation, and 223 were diagnosed after the 28th week of gestation. This study did not apply additional procedures for missing data, as the highest missing data rate was relatively low (3.8% for family history of diabetes). The characteristics of the study population and the incidence of gestational diabetes mellitus (GDM) are presented in Table 1. It shows that compared to the non-ICP group, the early-onset ICP group exhibited significantly higher levels of pre-pregnancy body mass index (BMI), parity, history of abortion, liver enzyme levels, and total cholesterol. The late-onset ICP group also showed a significant increase in pre-pregnancy BMI, parity, liver enzyme levels, and blood lipid levels when compared to the non-ICP group, but no significant differences were observed among pregnant women with a BMI of 28 or higher. Furthermore, no significant differences were noted in age, family history of diabetes, or history of macrosomia across early-onset or late-onset ICP groups when compared to the non-ICP group. Among individuals diagnosed with ICP between 24 to 28 weeks of gestation, parity, family history of diabetes, and liver enzyme levels showed significant differences when compared to the non-ICP group. However, other indicators such as history of abortion, and blood lipid levels did not exhibit the same significant differences as the other two ICP subgroups, possibly due to the smaller sample size of this group (only 82 individuals), which may have introduced greater bias.

**Table 1:** Baseline characteristics of cases.

Characteristics	All cases (*n* = 742)	Non-ICP (*n* = 315)	ICP (*n* = 427)							
			All ICP cases (*n* = 427)	*P*-value	Early-onset ICP (*n* = 122)	*P*-value	Onset in 24th-28th week (*n* = 82)	*P*-value	Late-onset ICP (*n* = 223)	*P*-value
Age (years)	29.84 ± 4.48	30.18 ± 4.19	29.60 ± 4.66	0.068	29.51 ± 5.22	0.165	29.20 ± 4.30	0.061	29.87 ± 4.46	0.411
Age ≥ 35 years	120 (16.17%)	44 (13.97%)	76 (17.8%)	0.161	27 (22.13%)	0.139	9 (10.98%)	0.478	40 (17.94%)	0.212
Pre-pregnancy BMI (kg/m^2^)	23.18 ± 3.34	22.49 ± 3.08	23.78 ± 3.44	**0.001**	25.47 ± 3.47	**0.038**	23.92 ± 3.48	**0.001**	23.19 ± 3.24	**0.001**
BMI ≥ 24 kg/m^2^	260 (35.04%)	92 (29.21%)	168 (39.34%)	**0.004**	54 (44.26%)	**0.003**	28 (34.15%)	0.385	86 (38.57%)	**0.001**
BMI ≥ 28 kg/m^2^	67 (9.03%)	20 (6.35%)	47 (11.01%)	**0.029**	20 (16.39%)	**0.001**	8 (9.76%)	0.283	19 (8.52%)	0.339
Multiparas	277 (37.33%)	179 (56.83%)	98 (22.95%)	**0.001**	12 (9.84%)	**0.001**	13 (15.85%)	**0.001**	73 (32.74%)	**0.001**
History of abortion	662 (89.22%)	267 (84.76%)	395 (92.51%)	**0.001**	118 (96.72%)	**0.001**	76 (92.68%)	0.062	201 (90.13%)	0.298
Family history of diabetes	42 (5.66%)	15 (4.76%)	27 (6.32%)	0.363	7 (5.74%)	0.676	9 (10.98%)	**0.036**	11 (4.93%)	0.928
Previous macrosomia	16 (2.16%)	8 (3.72%)	8 (1.87%)	0.537	3 (2.46%)	0.961	1 (1.22%)	0.474	4 (1.79%)	0.564
ALT (U/L)	32 (13,108)	24 (12,34)	73 (16,219)	**0.001**	85 (27,259)	**0.001**	37 (11,169)	**0.001**	84 (16,228)	**0.001**
AST (U/L)	32 (17,83)	25 (14,35)	54 (19,150)	**0.001**	65 (24,190)	**0.001**	33 (18,97)	**0.001**	63 (18,158)	**0.001**
TC (mmol/L)	5.95 ± 1.49	5.67 ± 1.15	6.16 ± 1.76	**0.001**	6.55 ± 1.53	**0.001**	5.57 ± 1.89	0.546	6.07 ± 1.87	**0.002**
TG (mmol/L)	4.77 ± 1.57	2.89 ± 1.32	3.17 ± 1.28	**0.004**	2.94 ± 1.16	0.714	2.95 ± 1.26	0.712	3.44 ± 1.32	**0.001**

BMI, Body mass index; ALT, Alanine aminotransferase; AST, Aspartate aminotransferase; TC, total cholesterol; TG, triglycerides. Bold values indicates *p*-value < 0.05.

Previous macrosomia was defined as birthweight >4,000 g.

Pre-pregnancy BMI was defined as the weight measured at the first prenatal visit (approximately 6 to 12 weeks of gestation) divided by the square of the height.

*p*-value represents comparison between all ICP patients or the subgroups of ICP patients and the non-ICP group.

To delve into whether ICP is a risk factor for GDM, we conducted a statistical analysis of GDM incidence rates across different ICP subgroups, comparing them with a non-ICP group. Given that our dataset only includes specific onset gestational ages for ICP patients but lacks this information for GDM patients, which is typically confined to the 24th to 28th week range, we cannot ascertain the sequence of GDM relative to ICP for patients diagnosed during this period. To counteract potential biases, we stratified ICP into early-onset (diagnosed before the 24th week of gestation) and late-onset (diagnosed after the 28th week of gestation) subgroups, excluding the analysis of 82 patients diagnosed with ICP between the 24th and 28th week in relation to GDM incidence. As shown in Table 2, compared to the control group (non-ICP group), the incidence of GDM among ICP patients is significantly higher (25.29% vs. 15.56%, *p* = 0.001). This trend is particularly pronounced in early-onset ICP patients, where the incidence of GDM is notably higher than in the control group (26.23% vs. 15.56%, *p* = 0.001). However, in late-onset ICP patients, the proportion of those also suffering from GDM was not significantly differ from the non-ICP group (21.52% vs. 15.56%, *p* = 0.076).

**Table 2:** GDM prevalence among ICP subgroups.

	GDM (*n*, %)	*p*-value
All cases (*n* = 742)	157 (21.16%)	
Non-ICP (*n* = 315)	49 (15.56%)	
All ICP cases (*n* = 427)	108 (25.29%)	**0.001**
Early-onset ICP (*n* = 122)	32 (26.23%)	**0.001**
Late-onset ICP (*n* = 223)	48 (21.52%)	0.076

*p*-value represents comparison between all ICP patients or the subgroups of ICP patients and the non-ICP group. Bold values indicates *p*-value < 0.05.

To pinpoint the risk factors linked to the development of GDM, we executed a univariate analysis. The regression model included age, pre-pregnancy, parity, levels of alanine aminotransferase (ALT), aspartate aminotransferase (AST), total cholesterol (TC), and triglycerides (TG) as continuous variables, alongside categorical variables like history of abortion, family history of diabetes and previous macrosomia. This analysis revealed that factors such as age, pre-pregnancy BMI, history of abortion, family history of diabetes, abnormal liver function (ALT and AST), elevated blood lipid levels (TC and TG), and early-onset ICP were associated with an increased incidence of GDM. To determine whether early-onset ICP (*n* = 122) was an independent risk factor for GDM, a multivariable logistic regression analysis was performed. This analysis adjusted for confounding effects of variables that showed significant differences between the groups, such as age, pre-pregnancy BMI, history of abortion, abnormal liver function, as well as factors considered classical risk factors for GDM from previous studies, including parity and family history of diabetes. After adjusting for these covariates, a significant correlation was found between early-onset ICP and increased GDM risk (OR 2.013, 95% CI 1.933–4.185) as shown in Table 3. Additionally, age, pre-pregnancy BMI, history of abortion, family history of diabetes, and ALT levels were also significantly associated with increased GDM risk. Nonetheless, no significant correlations were found between the risk of GDM and factors such as parity, previous macrosomia, or levels of AST, TC, and TG.

**Table 3:** Univariate and multivariate logistic regression of factors for GDM.

Variable	Univariate		Multivariate	
	OR (95% CI)	*p*-value	OR (95% CI)	*p*-value
Age	1.096 (1.051–1.142)	**0.001**	1.108 (1.004–1.212)	**0.001**
Pre-pregnancy BMI (kg/m^2^)	1.095 (1.036–1.158)	**0.001**	1.134 (1.082–1.309)	**0.038**
Parity	0.707 (0.478–1.046)	0.079	2.701 (0.773–9.444)	0.120
History of abortion	1.983 (1.966–4.071)	**0.045**	8.018 (1.073–9.820)	**0.043**
Family history of diabetes	3.094 (2.326–7.698)	**0.001**	7.561 (1.073–10.819)	**0.001**
Previous macrosomia	3.059 (1.143–8.182)	**0.034**	1.688 (0.519–5.484)	0.384
ALT (U/L)	1.002 (1.001–1.009)	**0.004**	1.005 (1.001–1.009)	**0.027**
AST (U/L)	1.002 (1.001–1.003)	**0.023**	1.004 (0.999–1.009)	0.121
TC (mmol/L)	1.162 (0.944–1.431)	0.155	1.157 (0.797–1.679)	0.444
TG (mmol/L)	1.127 (0.851–1.492)	0.401	0.869 (0.538–1.406)	0.569
Early-onset ICP	1.841 (1.252–2.708)	**0.002**	2.013 (1.933–4.185)	**0.001**

BMI, Body mass index; ALT, Alanine aminotransferase; AST, aspartate aminotransferase; TC, total cholesterol; TG, triglycerides; OR, Odds ratio; CI, Confidence interval. Bold values indicates *p*-value < 0.05.

Our next step involves subgroup analyses to evaluate whether the increased risk of GDM associated with early-onset ICP (diagnosed before the 24th week of gestation) exhibits heterogeneity across different populations. To enhance the credibility of these subgroup analyses, we have pre-specified the analysis plans for each subgroup. Consequently, even though factors such as parity and history of previous macrosomia did not show statistical significance in the multifactorial risk regression analysis for GDM, they remain significant risk factors for the development of GDM in pregnant women. These indicators are therefore included in the subgroup analysis to consider their interactions. Thus, we conducted stratified analyses based on several key variables to elucidate the interactions among various known factors influencing the development of early-onset ICP and GDM: age (≥35 or < 35 years), pre-pregnancy BMI (≥24 or < 24 kg/m^2^), parity (nullipara or multipara), history of abortion (present or absent), family history of diabetes or macrosomia (present or absent), and liver function (normal or abnormal). As shown in Table 4, early-onset ICP was linked to a heightened risk of developing GDM among women <35 years (OR 2.482, 95%CI 1.512–4.074), nullipara (OR 4.211, 95%CI 1.158–5.312), those without a family history of diabetes (OR 2.400, 95%CI 1.371–4.201), those without previous macrosomia (OR 2.769, 95%CI 1.623–4.723), and those with abnormal liver function (OR 2.961, 95%CI 1.327–6.611). This increased risk of GDM was significantly higher in early-onset ICP patients compared to non-ICP patients, regardless of abortion history (OR 1.890, 95%CI 0.064–5.917, OR 2.054, 95%CI 1.199–3.519). We then used the Breslow-Day test to evaluate the homogeneity of odds ratios (ORs) for ICP across different risk factor strata, and no significant differences were found, suggesting a consistent influence of early-onset ICP on the development of GDM across these strata.

**Table 4:** Incidence of GDM with respect to early-onset ICP in pregnant women, stratified by risk factors.

Factors	GDM (*n*, %)			*p*-value	OR	95% *CI*	*p*-value^a^
	All cases (*n* = 742)	Non-ICP (315)	Early-onset ICP (122)				
Age<35 years	92 (12.40%)	24 (7.62%)	21 (17.21%)	**0.001**	2.482	1.512–4.074	0.139
Age ≥ 35 years	50 (6.74%)	16 (5.08%)	11 (9.02%)	0.712	1.203	0.450–3.216	
Pre-pregnancy BMI <24 kg/m^2^	71 (9.57%)	25 (7.94%)	10 (8.20%)	0.439	1.366	0.620–3.008	0.665
Pre-pregnancy BMI ≥24 kg/m^2^	71 (9.57%)	15 (4.76%)	22 (18.03%)	**0.001**	3.529	1.626–7.660	
Nullipara	98 (13.21%)	21 (6.67%)	14 (11.47%)	**0.038**	4.211	1.158–5.312	0.269
Multiparas	44 (5.93%)	19 (6.03%)	15 (12.30%)	**0.044**	1.869	0.993–3.531	
History of abortion	133 (17.92%)	38 (12.06%)	30 (24.59%)	**0.011**	1.890	0.064–5.917	0.056
No history of abortion	9 (1.21%)	2 (0.63%)	2 (1.64%)	**0.009**	2.054	1.199–3.519	
Family history of diabetes	22 (2.96%)	6 (1.90%)	5 (4.10%)	0.181	3.750	0.540–6.045	0.665
No family history of diabetes	120 (16.17%)	34 (10.79%)	27 (22.13%)	**0.002**	2.400	1.371–4.201	
Previous macrosomia	7 (0.94%)	4 (1.27%)	1 (0.82%)	0.417	0.333	0.023–4.736	0.131
No previous macrosomia	135 (18.19%)	36 (11.43%)	31 (25.41%)	**0.001**	2.769	1.623–4.723	
Abnormal liver function	93 (12.53%)	10 (3.17%)	27 (22.13%)	**0.008**	2.961	1.327–6.611	0.187
Normal liver function	49 (6.60%)	30 (9.52%)	5 (4.10%)	0.690	1.232	0.442–3.435	

BMI, Body mass index; OR, Odds ratio; CI, Confidence interval. *p*-value represents comparison between early-onset ICP patients and the non-ICP group. ^a^*p*-values for interaction effect between each risk factor and ICP on GDM. Bold values indicates *p*-value < 0.05.

The above evidence suggests that early-onset ICP may predispose patients to an increased susceptibility to GDM. But does this susceptibility extend in the opposite direction? Is it possible that GDM patients are more prone to developing ICP? To delve into this intriguing question, we conducted a follow-up study. We specifically targeted patients who were not diagnosed with ICP before the 24th week of gestation but were diagnosed with GDM between the 24th and 28th weeks (*n* = 70). Our aim was to monitor these patients for the emergence of late-onset ICP (onset after the 28th week of gestation). The results indicate that there appears to be no significant association between GDM and the development of late-onset ICP (*p* = 0.279). However, patients with elevated AST levels and history of abortion are more susceptible to developing late-onset ICP, irrespective of GDM status (Table 5).

**Table 5:** Univariate and multivariate logistic regression of factors for late-onset ICP.

Variable	Univariate		Multivariate	
	OR (95% CI)	*P*-value	OR (95% CI)	*P*-value
Age	1.013 (0.946–1.085)	0.715	0.943 (0.853–1.042)	0.248
Pre-pregnancy BMI (kg/m^2^)	0.917 (0.832–1.011)	0.081	1.034 (0.916–1.168)	0.309
Parity	1.361 (0.648–2.856)	0.415	2.560 (2.468–7.688)	0.550
History of abortion	1.149 (1.085–1.929)	**0.048**	6.649 (1.744–9.392)	**0.009**
Family history of diabetes	0.899 (0.339–2.380)	0.831	1.147 (0.178–7.379)	0.885
Previous macrosomia	0.774 (0.145–4.144)	0.765	0.845 (0.517–5.887)	0.115
ALT (U/L)	1.003 (1.002–1.005)	**0.001**	1.002 (0.998–1.006)	0.258
AST (U/L)	1.004 (1.003–1.005)	**0.001**	1.006 (1.001–1.011)	**0.031**
TC (mmol/L)	1.279 (1.047–1.563)	**0.012**	1.138 (0.820–1.579)	0.439
TG (mmol/L)	1.368 (1.041–1.797)	**0.020**	1.113 (0.729–1.701)	0.620
GDM	1.240 (0.840–1.831)	0.279	0.770 (0.047–2.032)	0.269

Bold values indicates *p*-value < 0.05.

To explore the potential interplay between ICP and GDM on pregnancy outcomes, we conducted a comprehensive analysis of the outcomes among GDM patients, examining both those who also had ICP and those who did not. The patients were further stratified into three subgroups based on the gestational age at onset of ICP. The results indicated that GDM patients with ICP delivered significantly earlier than those without ICP (259 days vs. 274 days, *p* = 0.001). Additionally, they exhibited a higher rate of preterm birth (delivered before 37 completed gestational weeks) (45.10% vs. 7.50%, *p* = 0.001) and comparatively lower birth weights for their newborns (2,904 grams vs. 3,280 grams, *p* = 0.001). Similarly, ICP patients exhibit a higher incidence of small for gestational age (SGA) infants (18.63% vs. 2.50%, *p* = 0.013), while the proportion of large for gestational age (LGA) infants is comparatively lower than in patients without ICP (0.98% vs. 7.05%, *p* = 0.035). Notably, within the cohort of patients with ICP, the proportion of elective cesarean sections significantly exceeds that of patients without ICP (50.98% vs. 20.00%, *p* = 0.001). Specifically, the cesarean section rate among those diagnosed with early-onset ICP (59.38%) and those diagnosed with ICP between 24th and 28th weeks of gestation (63.64%) exceeds 50%. In contrast, among patients with late-onset ICP, the rate of elective cesarean sections is not too high (39.58%); instead, emergency cesarean sections are more common (60.42%). There were no fetal deaths in either group (Table 6).

**Table 6:** Pregnancy outcomes of GDM patients with or without ICP.

Characteristics	GDM without ICP (*n* = 40)	GDM with ICP							
		All ICP cases (*n* = 102)	*p*-value	Early-onset ICP (*n* = 32)	*p*-value	24th ≤ onset gestation<28 weeks (*n* = 22)	*p*-value	Late-onset ICP (*n* = 48)	*p*-value
GA at delivery, days	274 ± 8	259 ± 13	**0.001**	256 ± 14	**0.001**	256 ± 15	**0.001**	260 ± 11	**0.001**
Weeks +days	38 + 3	37 + 1		36 + 3		36 + 4		37 + 2	
Preterm birth	3 (7.50%)	46 (45.10%)	**0.001**	13 (40.63%)	**0.001**	11 (50.00%)	**0.001**	22 (45.83%)	**0.001**
Cesarean section	17 (42.50%)	73 (71.57%)	0.107	22 (68.75%)	**0.026**	20 (90.91%)	**0.001**	31 (64.58%)	**0.038**
Elective section	8 (20.00%)	52 (50.98%)	**0.001**	19 (59.38%)	**0.001**	14 (63.64%)	**0.001**	19 (39.58%)	**0.048**
Emergency section	9 (22.50%)	21 (20.59%)	0.802	13 (40.63%)	0.097	8 (36.36%)	0.242	29 (60.42%)	**0.001**
Birth weight, grams	3,280 ± 407	2,904 ± 502	**0.001**	2,837 ± 516	**0.001**	2,846 ± 550	**0.001**	2,962 ± 469	**0.002**
LGA, *n*	3 (7.50%)	1 (0.98%)	**0.035**	0 (0.00%)	0.114	0 (0.00%)	0.188	1 (2.08%)	0.225
SGA, *n*	1 (2.50%)	19 (18.63%)	**0.013**	6 (18.75%)	**0.021**	4 (18.18%)	**0.030**	9 (18.75%)	**0.017**
Apgar-1 score	9 (9–9)	9 (9–9)	0.117	9 (8–9)	0.039	9 (9–9)	**0.048**	9 (9–9)	0.417
Apgar-5 score	10 (10–10)	10 (10–10)	0.657	10 (10–10)	0.839	10 (10–10)	0.839	10 (10–10)	0.507
Postpartum hemorrhage	300 (200–300)	300 (200–300)	0.995	300 (200–300)	0.813	300 (200–300)	0.973	300 (200–300)	0.880

*p*-value represents among GDM patients, the comparison between all ICP patients or the subgroups of ICP patients and the non-ICP group. Bold values indicates *p*-value < 0.05.

**Figure 2:**
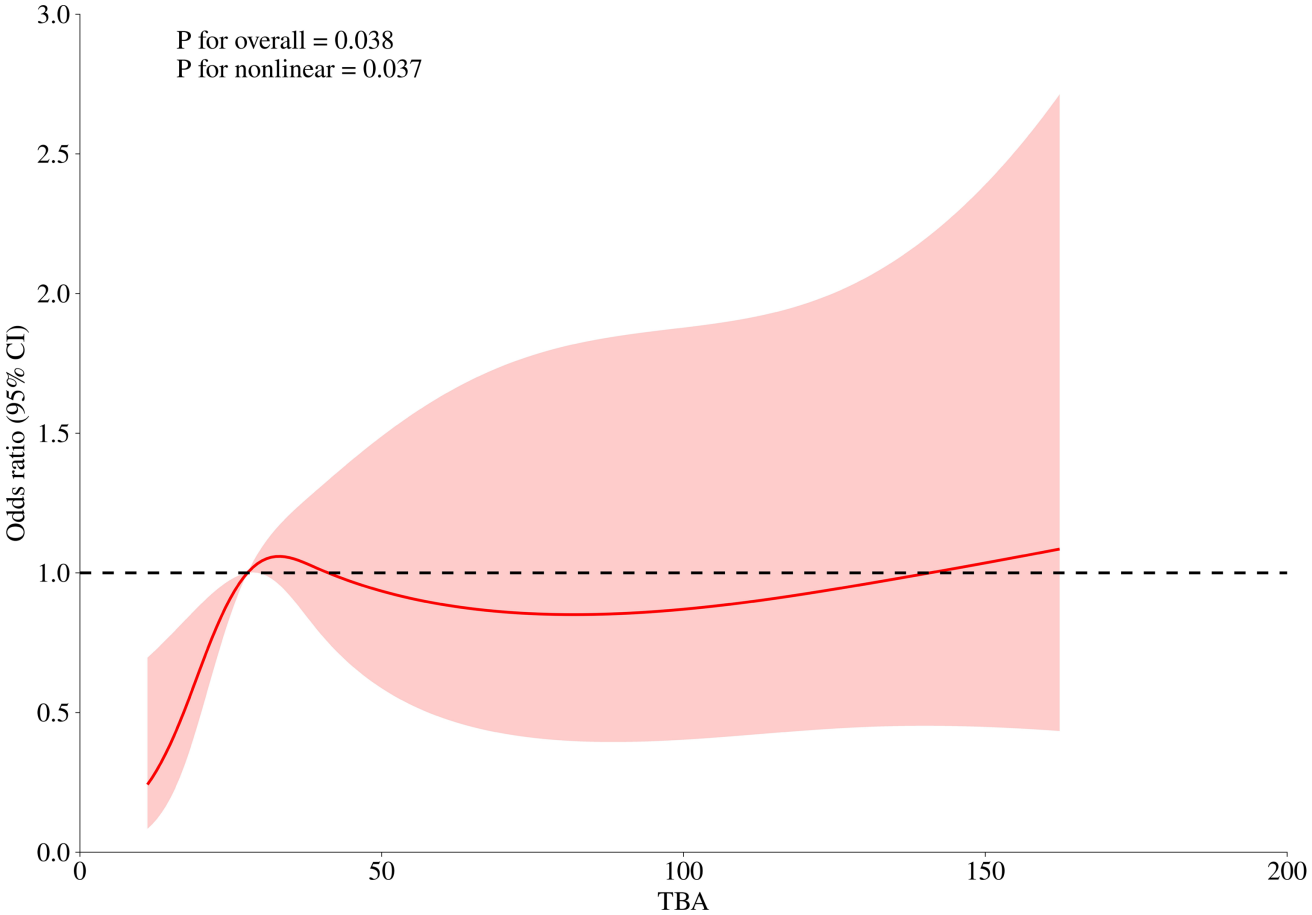
RCS regression analysis of TBA with GDM risk in early-onset ICP patients.

The level of TBA (first diagnosed) was significantly associated with GDM risk (OR 1.129 for each 1 μmol/L increase, [95%CI 1.089–1.279], *p* = 0.038) in early-onset ICP patients. Furthermore, a nonlinear association between the first diagnosed TBA levels and GDM risk was observed (*p* = 0.037). The RCS regression model revealed that elevated TBA levels were associated with an increased risk of GDM in a nonlinear fashion, and a TBA level of 27.75 μmol/L is identified as the reference point (Figure 2).

## Discussion

Intrahepatic cholestasis of pregnancy (ICP) and gestational diabetes mellitus (GDM) are two common pregnancy complications that pose considerable health challenges. The interplay between these conditions is believed to significantly influence pregnancy outcomes. Understanding the interplay between ICP and GDM is crucial, given the potential compounded risks both conditions pose to pregnancy outcomes. Establishing a clear link between these conditions will help in developing more effective management strategies for pregnant women suffering from these complications. However, the nature of this relationship remains elusive and has been a subject of debate in the literature.

Several studies have suggested an increased risk of GDM in patients with ICP, especially among those with severe cases or in twin pregnancies (7–10, 14–16). For instance, Martineau et al. found a positive correlation between ICP and the risk of GDM (7), a finding supported by Arafat and Dong (8). However, this result contradicts many other studies that support the hypothesis that women with ICP do not carry an additional risk of GDM. In a retrospective cohort study of a Danish population, Silja et al. found that patients with both ICP and GDM exhibited a distinct glucose metabolism profile compared to those with GDM alone, with no significant association between ICP and glucose control in GDM (12). Janina et al. found no significant differences in fasting glucose levels between ICP patients and healthy pregnant women, although postprandial glucose levels were significantly higher in ICP patients, albeit not reaching the GDM diagnostic criterion of 8.5 mmol/L (17). The timing of ICP onset, whether early or late in pregnancy, and its impact on GDM development is not well understood. Moreover, the adoption of the International Association of Diabetes and Pregnancy Study Groups (IADPSG) criteria in our study led to an increased diagnosis of GDM (13), which may have influenced the reported associations between ICP and GDM in more recent studies. Given these inconsistencies and the potential impact of the revised diagnostic criteria, our study aims to confirm the correlation between ICP and GDM and examine whether factors such as the timing of ICP onset, associated liver dysfunction, and TBA levels influence the risk of GDM.

The incidence of GDM is heightened in women who are predisposed to developing intrahepatic cholestasis of pregnancy, with this susceptibility being exacerbated following the onset of cholestasis (7). This notion aligns with our findings, which demonstrate that early-onset ICP (diagnosed before the 24th week of gestation) is an independent risk factor for GDM, increasing the risk more than the absence of ICP. When considering maternal age, early-onset ICP increased the risk of GDM among younger mothers under the age of 35 or in women without a family history of diabetes, similar to its effects in women with histories of macrosomia and pre-pregnancy BMI greater than 24 kg/m^2^. Furthermore, the risk of GDM escalated with early-onset ICP, regardless of parity. Although the Breslow-Day test did not show significant differences, it can be hypothesized that there are interactions between early-onset ICP and other maternal factors that result in varying risks of GDM among pregnant women. Our results indicate that the risk of GDM increases with age. Additionally, our study also indicated that the incidence of GDM increased in the pregnancy women with higher age, family history of diabetes or abnormal liver function, implying that increasing age, liver function and genetic factor are the important contributors to the development of GDM.

We observed a higher incidence of SGA infants among patients with GDM who also had ICP (including all ICP subgroups), compared to those GDM patients without ICP, which is consistent with the previously reported results (18). This suggests that ICP may be associated with impaired fetal growth, potentially due to the adverse effects of liver dysfunction and bile acid toxicity on placental function and nutrient transfer. The proportion of cesarean sections was significantly higher in the ICP group compared to the GDM without ICP group. This may be attributed to the increased risk of complications during labor and delivery in ICP patients, necessitating a more controlled delivery method. Within the ICP cohort, the rate of elective cesarean sections was notably high, particularly in early-onset ICP patients and those diagnosed between the 24th and 28th weeks of gestation. In contrast, patients with late-onset ICP had a lower rate of elective cesarean sections and a higher rate of emergency cesarean sections. This suggests that the timing of ICP onset may influence the mode of delivery, with early-onset cases more likely to be managed by elective cesarean sections.

Besides, our study also identified a significant nonlinear relationship between TBA levels and the risk of GDM occurrence in early-onset pregnancies. However, with an odds ratio (OR) of merely 1.129, it is not conclusive to infer that higher TBA levels (first diagnosed) will inevitably lead to GDM. Future research should focus on the implications of TBA levels exceeding the reference value of 27.75 μmol/L in the context of GDM risk in early-onset ICP patients.

Although researchers have noted the correlation between ICP and GDM and attempted to clarify their intrinsic links, the underlying mechanisms of this association remain unclear. A case–control study found that ICP occurs later than GDM (12), while another cohort study found that GDM occurs statistically significantly after ICP (7). In this study, patients with early-onset ICP (diagnosed before 24th weeks of gestation) were more likely to develop GDM, and this association remained statistically significant after adjusting for confounding factors. While our findings suggest a unidirectional susceptibility from early-onset ICP to GDM, the lack of a significant association between GDM and late-onset ICP challenges the notion of a bidirectional relationship. This could be due to the specific temporal dynamics of these conditions, where the pathophysiological mechanisms leading to ICP may not be sufficiently activated by GDM alone. The increased susceptibility of patients with liver function abnormalities to late-onset ICP is noteworthy. Liver function may plays a crucial role in the metabolism of estrogen and other substances that could influence the development of ICP (19).

Although some researchers noticed the correlation between ICP and GDM, and attempted to clarify the intrinsic links, the potential mechanism for this association is unclear. Several previous reviews and reports have indicated that the pathophysiology of GDM is associated with insulin resistance in a state of chronic inflammation (20). Patients with ICP may exhibit a chronic low-grade inflammatory state, where inflammatory factors such as tumor necrosis factor-alpha (TNF-α) and interleukin-6 (IL-6) may impair insulin signaling (21), thereby induce GDM (20). Additionally, a characteristic feature of ICP is elevated serum bile acid levels, which can increase the expression of the farnesoid X receptor (FXR) and disrupt the homeostatic pathways of the glucose balance system (22). Thirdly, hormonal changes during pregnancy significantly impact glucose metabolism. ICP might indirectly affect insulin activity by influencing hormone levels (23), such as estrogen and progesterone, thus inducing or exacerbating insulin resistance (24). Fourth, ICP patients often have liver function abnormalities, and the liver is a crucial organ in regulating blood sugar. Liver function abnormalities can lead to dysregulated glucose metabolism (25), thereby increasing the risk of GDM. Finally, environmental factors such as diet and lifestyle may also play roles in the development of ICP and GDM. For example, a high-fat diet could exacerbate the burden on the liver, affecting the synthesis and secretion of bile acids, thereby impacting glucose metabolism (26). The underlying mechanisms potentially triggered by ICP may lead to pathological effects that subsequently disrupt glucose homeostasis or insulin resistance. This could account for the observed findings in our study that early-onset ICP increases the propensity for GDM development, whereas late-onset ICP does not exhibit such an association. However, this does not imply that there is no biological connection between them. It is necessary to employ a larger sample size to further validate this relationship.

The strong connection we found between early-onset ICP and GDM revealed by our study has important implications for clinical practice. Firstly, it is crucial to detect and monitor liver function early in pregnant women, especially those with risk factors such as a history of giving birth to large babies (macrosomia) or a pre-pregnancy BMI greater than 24 kg/m^2^. This proactive approach can help in timely interventions, potentially reducing adverse pregnancy outcomes associated with GDM. Furthermore, the presence of ICP, particularly when diagnosed early in pregnancy, should lead to more detailed management strategies, including more frequent blood glucose monitoring and closer surveillance of both maternal and fetal health. In addition, education and counseling are crucial in this process, requiring communication of the link between ICP and GDM and their impact on maternal and fetal health to patients. Lastly, these findings have implications for research and policy, with future studies focusing on understanding the underlying mechanisms behind the association of ICP and GDM, and policymakers considering the impact of these findings on prenatal care guidelines and resource allocation. By integrating these research outcomes into clinical practice, we can better anticipate and manage the risks associated with ICP and GDM, ultimately improving maternal and fetal health outcomes.

The strengths of this study include a large cohort size and the use of multivariable logistic regression analysis to adjust for potential confounders, alongside an exploration of the associations between early-onset ICP and GDM. However, unavoidable limitations also exist. Firstly, as a retrospective study, recall bias may occur. Additionally, due to incomplete data, previous histories of ICP or GDM were not included. This may impact the authenticity of our results to some extent. Secondly, although our study demonstrated a positive correlation between early-onset ICP and GDM, its ability to infer causation is limited, necessitating a large-scale prospective study to further investigate this causal relationship. Thirdly, the lack of comprehensive data on ursodeoxycholic acid (UDCA) treatment prevents us from determining whether treated ICP affects the occurrence of GDM. Fourthly, we did not collect the specific gestational age at GDM diagnosis in our study, which makes women diagnosed between 24 to 28 weeks of gestation in an awkward position – we cannot distinguish whether GDM or ICP occurred first, which may reduce the validity of our study. Lastly, the statistical power of this study is 0.7615, slightly below the recommended standard of 0.8, mainly due to the relatively small sample size of the control group, which restricts our ability to detect small effect sizes. Despite this, our sample size is relatively large compared to similar studies, and the study design is rigorous with results thoroughly statistically analyzed. Future studies should consider increasing the sample size of the control group to enhance statistical power and further validate our findings.

## Conclusion

The study shows a positive correlation between early-onset ICP and the risk of GDM. Given the unknown pathophysiological mechanisms linking ICP and GDM, and the potential high prevalence of ICP in certain regions, further research is clearly needed in this field. Future studies should focus on the underlying mechanisms of ICP’s impact on gestational metabolism and explore how early diagnosis and intervention can reduce the incidence of GDM.

## Data availability statement

The raw data supporting the conclusions of this article will be made available by the authors, without undue reservation.

## Ethics statement

The studies involving humans were approved by the ethics committee of the Shanghai Public Health Clinical Center (2022-Y061-01). The studies were conducted in accordance with the local legislation and institutional requirements. Written informed consent for participation was not required from the participants or the participants’ legal guardians/next of kin because no intervention, anonymized data, public interest, minimal risk, standard care, legal compliance, historical data, no personal ID, non-experimental.

## Author contributions

YL: Data curation, Investigation, Software, Visualization, Writing – original draft, Writing – review & editing. ML: Supervision, Writing – review & editing. JL: Conceptualization, Methodology, Writing – review & editing. MS: Formal analysis, Project administration, Writing – review & editing. ZH: Data curation, Methodology, Software, Validation, Writing – original draft, Writing – review & editing. XZ: Methodology, Project administration, Supervision, Writing – original draft, Writing – review & editing.

## Footnotes

^1^ www.medsta.cn/software
